# BCGitis or genitourinary tuberculosis? Diagnostic dilemma following intravesical Bacillus Calmette–Guérin therapy: a case report

**DOI:** 10.1097/MS9.0000000000005227

**Published:** 2026-06-09

**Authors:** Aashish Lamichhane, Sadish Sharma, Prakriti Gautam, Shishir Gaire, Apil Pokhrel

**Affiliations:** a Provincial Center for Disease Control, Pokhara, Kaski, Nepal; b College of Medical Sciences Teaching Hospital, Chitwan, Nepal; c Province Hospital, Beni, Myagdi, Nepal; d Tribhuvan University Institute of Medicine, Kathmandu, Nepal

**Keywords:** BCGitis, genitourinary tuberculosis, intravesical BCG, non–muscle-invasive bladder tumor

## Abstract

**Introduction and importance:**

Intravesical Bacillus Calmette–Guérin (iBCG) therapy is a cornerstone in the management of non–muscle-invasive bladder cancer. Although generally safe, it can rarely lead to granulomatous inflammatory complications that closely mimic recurrent malignancy or genitourinary tuberculosis, particularly in tuberculosis-endemic regions.

**Case report:**

A 47-year-old male with type 2 diabetes mellitus and high-grade T1 non–muscle-invasive bladder carcinoma underwent transurethral resection followed by iBCG induction and maintenance therapy. One month after completing the third maintenance cycle, he developed hematuria and persistent lower urinary tract symptoms. Urinalysis revealed sterile pyuria and hematuria, and imaging demonstrated a new focal bladder wall lesion. Cystoscopic biopsy showed granulomatous inflammation with caseous necrosis without malignancy. Urine was positive for acid-fast bacilli and *Mycobacterium tuberculosis* complex on GeneXpert. Considering the recent iBCG therapy, a diagnosis of BCG-associated granulomatous cystitis (BCGitis) was made. BCG therapy was stopped, and anti-tubercular therapy with isoniazid, rifampicin, and ethambutol was initiated.

**Discussion:**

BCG-induced granulomatous cystitis represents a diagnostic challenge due to its clinical, radiological, and cystoscopic resemblance to bladder cancer recurrence and genitourinary tuberculosis. In tuberculosis-endemic settings, differentiating these entities is particularly difficult. Histopathology remains central to diagnosis, while molecular techniques may aid in species differentiation when available. Awareness of this complication allows timely diagnosis and avoids unnecessary invasive interventions or overtreatment.

**Conclusion:**

BCGitis is a rare but important complication of iBCG therapy. A high index of suspicion, combined with careful clinicopathological correlation, is essential for accurate diagnosis and appropriate management, especially in tuberculosis-endemic regions.

## Introduction

Urinary bladder cancer is among the most prevalent malignancies of the genitourinary tract, with urothelial carcinoma accounting for nearly 90% of all cases[1]. A significant proportion of patients present with non–muscle-invasive bladder cancer (NMIBC), which is characterized by a high risk of recurrence and progression[2]. The primary treatment for NMIBC is transurethral resection of bladder tumor (TURBT), which is performed cystoscopically under anesthesia[3].

HIGHLIGHTS

- Bacillus Calmette–Guérin–associated granulomatous cystitis (BCGitis) can closely mimic genitourinary tuberculosis after intravesical BCG therapy.
- Early-onset urinary symptoms after BCG warrant evaluation beyond routine cystitis.
- Histopathology is crucial to distinguish BCGitis from tumor recurrence.
- Tuberculosis-endemic settings increase diagnostic complexity after BCG therapy.
- Timely recognition prevents unnecessary oncological or surgical escalation.

Despite appropriate surgical management, NMIBC poses a significant risk of recurrence, with reported recurrence rates ranging from 15% to 61% annually, increasing to 31%–78% over a 5-year follow-up period[4]. To reduce disease recurrence, delay progression, and preserve the bladder, the standard care involves TURBT followed by intravesical Bacillus Calmette-Guérin (iBCG) therapy and/or chemotherapeutic agents[5].

Multiple studies have demonstrated superior recurrence-free survival among patients receiving adjuvant iBCG compared to TURBT alone ^[^6–8^]^. However, despite its proven efficacy and widespread use, iBCG therapy is linked to various side effects, ranging from minor local irritative symptoms to serious systemic problems. Local adverse effects of iBCG commonly include dysuria, urinary frequency and urgency, hematuria, and cystitis-like symptoms[9]. In contrast, serious complications related to BCG dissemination or exaggerated immune response are rare but potentially life-threatening. These include granulomatous prostatitis, epididymo-orchitis, osteomyelitis, pneumonitis, hepatitis, sepsis, and, less commonly, granulomatous cystitis[10].

We describe a case of a high-grade T1 NMIBC patient on maintenance iBCG therapy who developed BCG-induced granulomatous cystitis. This case emphasizes the difficulties in diagnosing post-BCG bladder lesions and the significance of considering BCG-related consequences when evaluating recurring bladder pathology. This article is written in accordance with the SCARE guideline[11].

## History

A 47-year-old male, a known case of type 2 diabetes mellitus, presented to the urology outpatient department on 5 July 2024 with complaints of gross hematuria, nocturia, urinary urgency, increased frequency, and a weak urinary stream. Baseline blood investigations at presentation were within normal limits. Ultrasonography (USG) of the abdomen and pelvis revealed a urinary bladder mass, described as a non-mobile hyperechoic lesion arising from the posterior wall of the urinary bladder. A contrast-enhanced computed tomography (CECT) scan of the abdomen and pelvis, performed on 6 July 2024, demonstrated a well-defined, heterogeneously enhancing endoluminal lesion measuring 55 × 52 × 42 mm, arising from the right posterolateral wall of the urinary bladder.

The patient underwent TURBT on 12 July 2024, and biopsy samples were sent for histopathological examination (HPE). HPE revealed a papillary urothelial neoplasm of high grade with superficial invasion up to the muscularis mucosa, consistent with TNM staging T1N0M0. Following TURBT, intravesical immunotherapy with BCG was planned, consisting of 6 weeks of induction therapy followed by 3 years of maintenance therapy, and was initiated on 29 July 2024. The patient initially tolerated the therapy well and showed clinical improvement. However, approximately 1 month after the completion of the third cycle of maintenance BCG therapy, on 19 September 2024, he developed recurrent hematuria, flank pain, and lower urinary tract symptoms (LUTS). Further evaluation was undertaken, and laboratory investigations revealed the following (Table 1).

**Table 1:** Laboratory blood and urine findings.

Test	Result	Units	Reference range
Complete blood count (CBC)			
RBC	4.49	million/ µL	4.5–5.5
WBC	11 540	cells/mm^3^	4000–11 000
Platelets	335 000	cells/mm^3^	150 000–450 000
Hemoglobin	13.1	g/dL	12–18
Renal function test (RFT)			
Serum creatinine	0.93	mg/dL	0.5–1.4
Urea	25.53	mg/dL	15–40
Sodium	138	mmol/L	135–145
Potassium	4.38	mmol/L	3.5–5.0
Erythrocyte sedimentation rate (ESR)	32	mm/h	0–20
Urine routine microscopy (Urine RME)			
Color	Light yellow		
Albumin	Positive (+)		
Sugar	Trace		
pH	Acidic		5.0–-8.0
Specific density	1.007		1.005–1.030
Red blood cells	Plenty	/High power field	0–2
Pus cells	Plenty	/High power field	0–5
Epithelial cells	1–2	/High power field	0–5
Casts	Nil		

Based on the urine routine microscopy findings, a urine culture was sent. Empirical antibiotic with intravenous levofloxacin 500 mg once daily and other symptomatic management was initiated. However, the urine culture subsequently returned sterile, and antibiotics were withheld.

**Figure 1.**
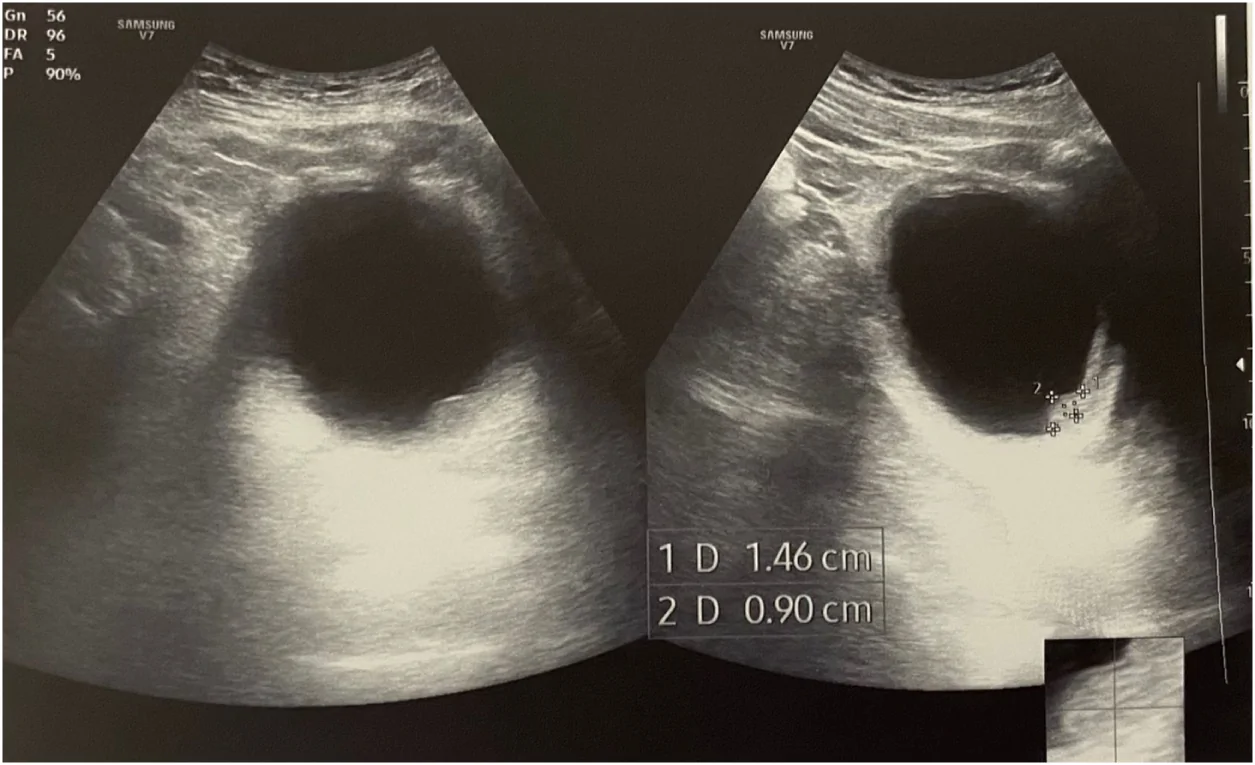
USG showing echogenic foci measuring 14 × 9 mm in the left posterolateral wall of the urinary bladder.

A USG performed on 23 November 2025 revealed a 14 × 9 mm echogenic focus in the left posterolateral wall of the urinary bladder (Fig. 1). Cystoscopic evaluation was conducted and revealed a small mucosal lesion on the left lateral wall of the urinary bladder, from which a tissue sample was obtained and sent for HPE.

HPE demonstrated granulomatous inflammation with caseous necrosis. Further microbiological evaluation was conducted using a urine sample sent for acid-fast bacilli (AFB) and GeneXpert-MTB/Rif testing. Both tests were positive for *Mycobacterium tuberculosis* complex, with no resistance to first-line anti-tubercular drugs detected. The sputum GeneXpert-MTB/Rif test was negative for MTB.

In the context of recent iBCG therapy, clinical features, histopathological findings, and microbiological results, the patient was diagnosed with BCG-associated granulomatous cystitis (BCGitis). iBCG therapy was withheld, and the patient was initiated on 6 months of isoniazid, rifampicin, and ethambutol with close clinical follow-up.

## Discussion

This case underscores a rare yet clinically significant complication of iBCG therapy. The primary challenge lies in the diagnostic dilemma between genitourinary tuberculosis, recurrence of bladder tumor, and BCG-induced cystitis. The clinical, histopathological, radiological, and microbiological profiles of BCGitis and genitourinary tuberculosis are largely indistinguishable, with only subtle clinical differences separating the two conditions. This marked overlap complicates accurate diagnosis and potentially delays timely and appropriate management.

iBCG immunotherapy remains the established gold-standard adjuvant therapy for patients with high-risk and selected intermediate-risk NMIBC[5]. To ensure maximal treatment efficacy, adjuvant BCG should be administered as maintenance therapy for at least 1 year in patients with intermediate-risk disease and for up to 3 years, when tolerated, in those with high-risk disease^[^5,12^]^. Although the precise mechanism underlying the anti-tumor effects of iBCG therapy remains incompletely understood, proposed mechanisms include direct interaction with urothelial and malignant cells, stimulation of innate immune responses, and induction of both BCG-specific and tumor-directed adaptive T-cell immunity[13].

Bacterial urinary tract infection was initially considered due to the presence of pyuria and hematuria; however, sterile urine cultures effectively excluded this possibility. Tumor recurrence was another concern, prompting cystoscopic evaluation, but negative HPE ruled out malignancy. The absence of recent catheterization made traumatic cystitis unlikely. HPE demonstrated granulomatous inflammation with caseous necrosis, and positive urine for AFB staining suggested *M. tuberculosis* complex infection. Nevertheless, the lack of systemic signs and symptoms, along with the recent history of iBCG instillation, argued against active genitourinary tuberculosis.

The clinical spectrum of BCG-associated disease reflects the nature and extent of underlying immune dysregulation. Disseminated disease indicates a significant failure of cell-mediated immunity required for effective control of intracellular pathogens, whereas localized manifestations are more likely to arise from an intact but exaggerated inflammatory response[14]. Although the precise mechanism of systemic infection following iBCG instillation remains unclear, local inflammation-induced disruption of the bladder urothelial barrier is thought to facilitate hematogenous or lymphatic dissemination of BCG organisms. This hypothesis is supported by reports demonstrating elevated urinary levels of IP-10, IL-6, and IL-8 in patients who develop iBCG-related adverse events[5].

Management of BCGitis is tailored to the severity of toxicity, ranging from temporary postponement or cessation of intravesical instillations to the initiation of anti-tubercular therapy for up to 6 months[15]. As an attenuated strain of *Mycobacterium bovis*, BCG exhibits intrinsic resistance to pyrazinamide, reflecting its known species-specific drug susceptibility profile. The most commonly employed therapeutic regimen comprises isoniazid and rifampicin administered for 6 months, with ethambutol added in the initial 2-month intensive phase, with or without fluoroquinolones[16]. In this case, BCG immunotherapy was promptly discontinued, and the patient was started on a 6-month course of isoniazid (H), rifampicin (R), and ethambutol (E).

BCG-induced cystitis alone is a rare entity in itself, with only a few cases reported till date, most of which included disseminated disease. A case reported by Selmi *et al* described renal granulomatosis with polyangiitis following iBCG therapy, with HPE revealing non-caseating granulomas, unlike our patient. Treatment involved anti-tubercular therapy alongside corticosteroids and cyclophosphamide, highlighting a difference in therapeutic strategy compared with our management approach, where we opted for anti-tubercular therapy alone[17]. Skowroński *et al* reported a patient with bladder carcinoma who, similar to our case, received iBCG therapy but subsequently developed miliary tuberculosis, diagnosed on chest radiography and confirmed by a positive sputum culture for *M. bovis*[18]. This presentation differs markedly from our case; however, both patients were managed with anti-tubercular therapy, underscoring the diverse spectrum of systemic manifestations associated with iBCG instillation.

This report is limited by the unavailability of polymerase chain reaction-based molecular speciation to distinguish *M. bovis* from *M. tuberculosis*, and by the lack of long-term clinical and oncological follow-up at the time of manuscript preparation.

## Conclusion

BCGitis is a rare but clinically important complication of iBCG therapy that can closely mimic recurrent urothelial carcinoma or genitourinary tuberculosis. Early-onset, persistent urinary symptoms following BCG instillation should prompt thorough evaluation, including cystoscopic biopsy and microbiological testing. Recognition of this entity is particularly crucial in tuberculosis-endemic settings to avoid misdiagnosis and inappropriate treatment escalation.

## Data Availability

No data were used or analyzed for the research work described.
